# The patient voice in research—evolution of a role

**DOI:** 10.1186/s40900-016-0020-4

**Published:** 2016-02-22

**Authors:** Dianne S. Johnson, Mary T. Bush, Susan Brandzel, Karen J. Wernli

**Affiliations:** Group Health Research Institute, 1730 Minor Ave, Suite 1600, Seattle, WA 98101 USA

**Keywords:** Patient engagement, Patient-centered, Comparative effectiveness research, Clinical studies

## Abstract

**Plain english summary:**

Engaging patients in research studies is becoming more common because it makes research and its results more relevant for patients. It is important to understand the best ways for patients and researchers to work together. Patients who are included as active partners in research can provide useful input on what it is like to work on a research team but very little has been written about this from the patient’s perspective. As patient partners and researchers on a breast cancer study, we share our experience to develop a patient-centered project and the inclusion of patient collaborators as scientific experts. Over time, the role of the patient partner has developed to include unanticipated roles and responsibilities. We use our experience to share how the patient voice can affect the execution of a research study and to provide a model for meaningfully engaging patients in research.

**Abstract:**

Engaging patients in research studies is becoming more common because it makes research and its results more relevant for patients. It is important to understand the best ways for patients and researchers to work together. Patients who are included as active partners in research can provide useful input on what it is like to work on a research team but very little has been written about this from the patient’s perspective. As patient partners and researchers on a breast cancer study, we share our experience to develop a patient-centered project and the inclusion of patient collaborators as scientific experts. Over time, the role of the patient partner has developed to include unanticipated roles and responsibilities. We use our experience to share how the patient voice can affect the execution of a research study and to provide a model for meaningfully engaging patients in research.

## Background

Patient partnerships in research have grown in popularity [1, 2]; however, little has been written about the relationship to research from the patient’s perspective, particularly within U.S. settings. We are patient partners (DJ, MB) and researchers (SB, KJW) on a breast cancer study entitled Surveillance Imaging Modalities for Breast cancer Assessment (SIMBA), funded by the Patient-Centered Outcomes Research Institute (PCORI) [3]. This study compares the effectiveness of mammography to mammography plus breast MRI for breast cancer surveillance in women with a personal history of the disease. The structure of our project includes a group of researchers, similar to most research projects; a stakeholder panel of 12 including local practitioners in different specialties, patients, and representatives from the cancer support and advocacy community; and a patient advisory board of 12 local women who have completed breast cancer treatment. Combined, our work aims to produce relevant results to all end-users within the breast imaging community. In this commentary, we discuss the evolution of our roles and the patient partners’ impact on SIMBA research activities to fill the knowledge gap in patients’ perspective to equitable engagement in research.

### Engagement as partners

Patient Partners: Prior to the start of the project, we saw an announcement for upcoming discussion on breast imaging in women after treatment for breast cancer. We participated in local focus group discussions about breast imaging, and were subsequently asked to join the project as patient partners in the study. We initially thought our role would be as volunteers; we would give our opinions and have an opportunity to see firsthand how research is conducted. However, the Principal Investigator’s (PI) (KJW) vision was very different. In our first meeting, she asked us to be co-investigators, meaning we would have a say in all aspects of the study and be paid for our time. She sketched out how she envisioned the organizational structure, placing us in the center of all research activities. Our role was to listen carefully to all team members and represent the patient experience (not just our own) throughout all components of the project. At first, we felt unsure about how we would accomplish this work, how much time it would take, and if we were qualified. However, through our experience with a supportive team and clear patient inclusion, we have been able to demonstrate the value of patient voices in research discussions and made a successful collaboration.

Researchers: We envisioned from the beginning that patient voices would be relevant to all aspects of our research, and included two patient partners in our proposal as co-investigators, to serve in the same manner as other colleagues with their subject matter expertise as patients. We were unclear how their role would be received by the broader research team. Over time, they had been integrated and welcomed as active members of the scientific team. Through study meetings, the patient perspectives have shaped the presentation of our research results, serving as a reminder that our data must represent and contribute to real women with real lives. For example, when understanding the impact of additional biopsy procedures, our patient partners shared the anxiety associated with undergoing the procedure, especially for women already treated for breast cancer and ensured that these emotions were not downplayed as we executed our analytic comparisons of breast imaging.

### Support and resources

Patient Partners: Our first task was participation in monthly scientific research team conference calls. It was daunting to join a group of scientists with high levels of expertise and familiarity with one another. To our ears, the conversation was filled with acronyms and unfamiliar terms. When we expressed our confusion, the SIMBA Project Manager (SB) created a useful glossary for us [4]. She also facilitated our completion of the required training course in human subjects research, a necessity for our role as Co-Investigators, and helped us better understand research.

The PI and Project manager met with us regularly since the study started. They took time to get to know our skills and abilities and invited us to take more initiative at meetings. We worked together to develop agendas and debriefed after the meetings. At the first stakeholder panel, we were asked to present findings from the patient advisory board meeting, sharing the patient perspective. While it was intimidating to present to physicians and researchers, it was the building block to create a collaborative group between ourselves and the scientific experts, and to be recognized as experts in the patient experience.

Researchers: Engaging patients takes effort to support their development and incorporation into research culture. We worked to prepare them in advance of study activities so they felt confident in their role. We also discussed study activities after our team meetings, so that we could hear their perspectives and answer any outstanding questions that may have arisen. We have now integrated patient partners into leading sections of meetings, helping to develop meeting agendas, and speaking on panels of patient engagement. In addition to the activities discussed above, we also engage our patient partners in team building activities (i.e., an annual hike), to enhance personal connection that can sometimes be missing in research teams. To document our engagement of patient partners, we always take meeting minutes, send biannual newsletters to our patient advisory board, and conduct after meeting surveys online, to understand the impact of engagement.

### New opportunities

Patient Partners: The PI consistently demonstrated flexibility and willingness to take risks and opened new opportunities for us to engage in the research. For example, a scientific team member suggested we join the PI and Project Manager to conduct project focus groups across the U.S., a deviation from the original research plan. Although the travel required changing our regular work schedules and being away from our families, we had the opportunity to engage more women with prior breast cancer and hear different patient perspectives. Throughout the focus group discussions, it was evident that having patient partners present instilled added trust and confidence in the women participating that their voices would be heard and taken seriously. For example, before one focus group, one of us greeted a participant who was nervous when she arrived. By sharing that we also were patients and explain what would happen during the focus group, the nervous participant was reassured and ready to contribute when the discussion began. In another focus group, one of us made a gentle comment about some very poignant “doodles” a participant drew during the group. The woman then opened up about the painful experience of mammograms, leading to an important discussion of anxiety about surveillance imaging and ways of coping.

During the first in-person research team meeting at the end of the first year, there seemed to be a shift in how we were received by other researchers. As discussions emerged throughout the day, scientific team members asked directly for our input on the patient perspective for many issues, which was different than our experiences from conference calls. We felt like we had become full members of the team. The same collegiality also developed at the stakeholder panel meeting, which included robust give and take conversations.

Another surprising opportunity arose about a year into our study. We were asked to participate in two panel discussions on patient engagement in research in front of broad audiences [5]. While we were excited and wanted to share our experiences, we were faced with a decision about how public we wanted to be with our health status. How would it feel knowing that anyone could search our name online and see we had breast cancer? Although this idea made us feel more vulnerable, we each chose to move forward feeling that the potential benefits outweighed the risks. We also had requests for media interviews as a result of this exposure and we were provided with media training on how to talk to reporters. This proved immensely helpful and eliminated possible difficulties we might have encountered during the interviews.

**Fig. 1:**
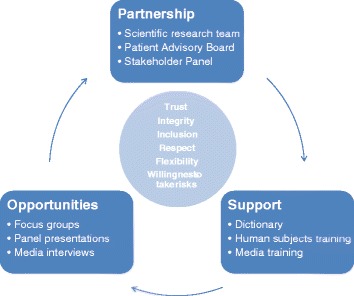
A model of patient engagement to support patient partnerships with researchers

The growth in our role with SIMBA project activities is attributable to leadership’s facilitation in our integration into the project. The PI and Project Manager have been committed to our inclusion from the beginning. Their integrity has inspired our trust and given us the courage to take risks in our expanded responsibilities. Their example has influenced members across the SIMBA team and allowed us to be integral contributors to the research. Based on our experience, we present a model to help guide successful collaboration between researchers and patients (Fig. 1). Key elements of the model include partnership, support, and opportunity. These elements operate in a cycle that helps continuously build a team, and in turn strengthens our partnership and leads to deeper engagement. In the center of the model are core values that all parties need to bring to the table, including trust, integrity, inclusion, respect, flexibility, and willingness to take risks.

Researchers: Our Patient Partners have had an opportunity to both influence qualitative research and observational study analysis, in unexpected ways. We had not initially planned for patient partners to attend focus groups with women with prior breast cancer. However, when we hosted our first focus group, it became quite evident how important their role was to our team. Patient partners were able to make a connection with the participants that helped the research team and participants develop a sense of trust almost immediately that would have been more difficult to establish in their absence. Further, we were criticized in our grant application for not specifically addressing mortality as a main outcome in our analysis. However, our patient partners helped us refute that mortality is not the only important outcome to patients, especially when chances of it are relatively low, and other outcomes such as unnecessary diagnostic procedures were very important to patients. The inclusion of patient perspectives in the conduct of research can be new for some researchers, especially in settings with established collegial relationships. Because of these relationships, the incorporation of new members can have challenges but these can be overcome with patience, respect of the team members, and in-person meetings.

### Patient impact

#### Patient partners

Our presence has helped shape SIMBA because we represent the human face of research, and we are a reminder that the study data represent real women who will be affected by the surveillance imaging they receive. SIMBA scientists are willing to listen to the patient voice we bring and incorporate it in the project, from including us in focus groups to brainstorming opportunities for meaningful dissemination of results*.* Our attendance at all SIMBA meetings allows us to serve as messengers and translators of the project’s progress as well as share questions and opinions between very different members of the team. Conversations in focus groups and the advisory board are filled with important themes from patients. These opinions influence how patients would prefer to receive information needed for decision making in their care plans and identify the need for additional communication between patients and providers about their survivorship care and receipt of breast imaging. We have experienced firsthand how patient engagement in research can provide new ideas to improve health care and ensure it focuses on patient needs.

#### Researchers

By having the patient voice at the research table, we are able to think about results as being meaningful to patients not just data for academic journals. With patient partners, our discussions have changed to make us more cognizant of how patients might be directly affected by the research results generated and how to use the information. Conducting the research this way raises the question of how the research changes if patient partners were not involved in this research program. At the end of the study, the production of the statistical results in academic journals would have likely been similar; however, our discussions and interpretation of the results have been markedly changed by the patient partners, as has our planning for how the results will be conveyed to all end-users, specifically patients. We recommend the inclusion of patient partners at least as co-investigators with their expertise in the patient experience to be collaborators in clinical research.

## Conclusions

Effective patient engagement is a time consuming process and significant investment. The research team and patient partners need to be open to the risks and be flexible in this work together. Mutual trust and integrity are key components to keep open conversation flowing and offer the possibility of allowing the patient voice to impact research studies, which can be incredibly valuable in providing end-use of research results. Future studies with a direct impact on patient-centered outcomes research would directly benefit from engagement with patients as full-team members in their research programs.
